# Food intake assessment and anthropometric characterisation of teenage gymnasts

**DOI:** 10.1080/07853890.2021.1896081

**Published:** 2021-09-28

**Authors:** Marcela Holanda, Patrícia Lavado, Filipa Vicente, Renata Ramalho, Paula Pereira

**Affiliations:** a Instituto Universitário Egas Moniz (IUEM), Egas Moniz Cooperativa de Ensino Superior, Caparica, Caparica, Portugal; b Centro de Investigação Interdisciplinar Egas Moniz (CiiEM), Egas Moniz Cooperativa de Ensino Superior, Caparica, Caparica, Portugal; c Grupo de Estudos em Nutrição Aplicada (GENA), Instituto Universitário Egas Moniz (IUEM), Egas Moniz Cooperativa de Ensino Superior, Caparica, Portugal

## Abstract

**Introduction:**

Food and nutrition have a critical role in health, sports performance but also in a proper growth a development when we consider young athletes [1,2]. In addition to the increased energy needs, there is a special concern in ensuring adequate intakes of specific micronutrients such as calcium and iron [3]. The aim of the present study was to evaluate the food intake of a group of teenager gymnasts and to compare energy, macronutrient and micronutrient intake with recommendations for this age group.

**Methods:**

In this observational study, the teenager gymnasts had answered two 24 h dietary recall applied three days apart. The sample included 66 participants 11–17 (14.74 ± 1.79) years old and 26 (39%) were male. Participants were weighted in a 100 g digital sacale (Beurer GS-10) and had their height measured in a stadiometer (SECA213). Body fat had been assessed through skinfold measurement using a INNOVACARE Cescorf through Durnin & Womersley formula which included 5-site measures (bicep, tricep, subscapula, suprailiac and abdominal).

**Results:**

Results had shown that 15% were overweight and only 3% were obese, 39% of the sample had normal values of body fat. The average energy intake was 1660 ± 621 kcal while the calcium average intake was 652 ± 307 mg, significantly lower than the recommended value (*p* = .02). The average Iron intake in female athletes was 7.68 ± 2.81 mg, also significantly lower than the recommended (*p* = .03). In this sample 23 participants (35%) of the young athletes did not had any dairy portion and 47 (72%) did not had any portion of vegetables daily.

**Conclusions:**

Data of this sample had shown a very low calcium intake in the participants and also a low iron intake in female individuals. Considering the age group, these results reinforce the importance of the nutritionist role in sports specially in young athletes in order to improve not only individual choices but also nutritional knowledge.

## References

[1] Meyer F, O'Connor H, Shirreffs SM. Nutrition for the young athlete. J Spors Sci. 2007;25(sup1):S73–S82. 10.1080/02640410701607338 18049985

[2] Jeukendrup A, Cronin L. Nutrition and elite young athletes. Med Sport Sci. 2011;56:47–58. 21178366 10.1159/000320630

[3] Purcell L. Sport nutrition for young athletes. Paediatr Child Health. 2013;18(4):200–202. 24421690 10.1093/pch/18.4.200 PMC3805623

